# Debate: Conversational AI and young people's mental health—friend or foe? A case for designing conversational AI as a bridge to youth mental health support

**DOI:** 10.1111/camh.70105

**Published:** 2026-07-02

**Authors:** Jessica L. Schleider, Rob Morris

**Affiliations:** ^1^ Northwestern University Chicago Illinois USA; ^2^ Koko USA

**Keywords:** Adolescence, Intervention, Mental health

## Abstract

Conversational AI is now deeply embedded in adolescents' mental health help‐seeking and emotional lives, creating both risks and a rare population health opportunity. This debate piece argues that the key question for researchers and implementers to consider is no longer *whether* adolescents should use general‐purpose chatbots for mental health support, but rather *how* scientists, regulators, and technology companies should shape these systems to reduce harm and promote constructive action. At present, built‐in chatbot responses to user expressions of distress often emphasize detection, refusal, or crisis referral, strategies that may protect developers but can fail to meet adolescents' immediate needs. At the same time, open‐ended, pseudo‐therapeutic interactions with untested agents can reinforce risk, dependency, and inaccurate or harmful beliefs about mental health and help‐seeking. We propose that general‐purpose conversational AI platforms are well‐positioned to function as *bridges* to evidence‐based support, rather than as replacements for formal therapy or treatment. Brief, bounded interventions, including digital single‐session interventions, offer a promising model for responding to moments of need while preserving adolescent agency. Ethical AI design should prioritize safeguards, empirical testing, and pathways to evidence‐based care.

The rapid proliferation of conversational AI agents has made them an unignorable feature of adolescents' emotional lives. Across the United States, 20.5% of 10‐ to 12‐year‐olds, 42.0% of 13‐ to 14‐year‐olds, and 50.4% of 15 –to 17‐year‐olds used generative AI apps in 2025 (Maheux et al., 2026), with a sizable minority using these tools for hours each day. Adolescents use these systems to ask questions they may hesitate to raise with parents, teachers, or peers; to explore their identities and relationships; and to process difficult emotions, often in moments of acute need (Ajmani et al., 2026). Given this reality, debates around adolescents' AI use should not center on whether AI tools should shape teens' help‐seeking and mental health (they already do), but what roles we should build them to play, within what guardrails, and toward what ends for population health.

Conversational AI holds clear potential for harm. General‐purpose chatbots (e.g. ChatGPT) were not developed as evidence‐based mental health interventions. Their responses can be inaccurate, inconsistent, and miscalibrated to psychological risk. They may miss indirect expressions of suicidality, reinforce distorted beliefs, offer unsafe guidance, or foster maladaptive reassurance‐seeking, particularly among adolescents with existing anxiety or depression symptoms (Golden, & Aboujaoude, 2026). Some systems respond to mental health disclosures with generic refusals or crisis referrals; such responses may reduce liability for developers, but they do not necessarily help users. Some users have expressed that refusals communicate dismissal and may discourage further help‐seeking (Ajmani et al., 2026). Recent work has argued against this avoidance‐based approach and toward a more empowerment‐oriented model, in which AI supports users' agency, de‐escalates risk, and helps identify feasible next steps (Kaveladze et al., 2026a). A related concern is that general‐purpose chatbots are optimized to produce replies that feel helpful, agreeable, and engaging in the moment, not necessarily to catalyze future improvements in emotion regulation, coping, or help‐seeking. In mental health contexts, this may create a mismatch between what feels comforting immediately and what is reliably helpful over time.

Simultaneously, purely negative framings miss an opportunity inherent to these tools: their unprecedentedly rapid adoption. General‐purpose chatbots have achieved uptake rates rarely seen among digital mental health tools, likely because conversational AI offers frictionless, on‐demand interaction with an empathetic‐seeming entity that requires no interpersonal self‐disclosure (i.e. no risk of judgment or unwanted external intervention). Adolescents, in particular, highly value (and seldom have access to) privacy and autonomy when seeking mental health support (Schleider, & Fox, 2025); simultaneously, many face barriers to accessing formal services, including high costs, long waitlists, stigma, and clinician shortages. Indeed, more than half of US youth with mental health needs access no support (Whitney, & Peterson, 2019). It therefore follows that youth would turn to these flexible, accessible tools for help. Their widespread naturalistic uptake also creates a rare population health opportunity to connect high‐need adolescents, at scale, to supports that are more immediate, lower‐burden, and more likely to be used than many alternatives (e.g. existing mental health apps requiring separate downloads, parental involvement, or paid subscriptions). We argue that this opportunity creates an ethical necessity—both for researchers and AI companies—to systematically examine, de‐risk, and improve the effectiveness of general‐purpose conversational agents to support adolescent mental health.

The key challenge, then, is how to keep adolescents safe while still providing meaningful in‐the‐moment support via conversational agents. To date, efforts have either gone untested or fallen short of this goal. As one example, safety features for younger users, such as ChatGPT's “teen accounts” wherein parents are notified when youth safety concerns are detected, may prove beneficial—but only if they are carefully calibrated to actual risk and implemented transparently. If built without clear communication to and partnership with young users, such tools may inadvertently undermine autonomy, reduce trust, and discourage future disclosure or help‐seeking. Separately, in the United States, myriad policies meant to enhance LLM safety require chatbots to continually remind users that it is not a human; however, there is no clear evidence that problematic LLM use results from beliefs that LLMs are human, nor are reminders clearly linked to safer interactions. Perhaps most consistently, built‐in responses to risk disclosures in chatbot interactions tend to emphasize detection, refusal, and redirection to crisis lines or in‐person psychotherapy (which many adolescents cannot access independently). Although these strategies may reduce legal exposure, they do not constitute evidence‐based support and may fail to meet adolescents' immediate needs in moments of distress. Redirections alone do not resolve the underlying reality that many users will continue to disclose needs and seek help via chatbots. A responsible population health response requires us to assume continued use and shape design features accordingly (Ajmani et al., 2026).

Supports embedded within conversational AI must be usable in the moment; reliably better than nothing (i.e. evidence‐based relative to realistic control conditions; not subject to idiosyncrasies of different models); and respectful of young people's personal developmentally appropriate drives to exercise agency (Schleider, & Fox, 2025). As such, we argue that the optimal role for conversational AI in adolescent mental health is as a bridge to evidence‐based support, not as a “therapist” itself. Built responsibly, conversational AI could serve as a powerful touchpoint at early stages of help‐seeking and disclose that recognizes distress, preserves agency, mitigates immediate harm, and helps youth take the best next step for which they are ready. This example reflects how many digital help‐seekers already interface with conversational agents. Ajmani and colleagues (2026) found that “people who turn to conversational AI agents during mental health crises do not experience these tools as their final or only sources of care. Instead, they turn to these tools as bridging supports—spaces that help people navigate the overwhelming gap between extreme distress and healthy coping.”

Research on digital single‐session interventions suggests that brief, embedded supports can function effectively in this bridging role. Across studies conducted in partnership with online platforms, we have found that very brief interventions can improve immediate outcomes and increase uptake of further support. In one randomized study, users who received an enhanced crisis‐response single‐session intervention showed greater reductions in hopelessness and were more than twice as likely to use the resources provided than users who received a standard crisis‐line response alone (Cohen, Dobias, Morris, & Schleider, 2023). In another study, 10‐minute single‐session interventions embedded in a social media platform were completed thousands of times, received strong acceptability ratings, and were associated with short‐term improvements in hopelessness, self‐hate, and desire to stop self‐harm (Dobias, Morris, & Schleider, 2022). These findings align with randomized trial evidence showing that digital SSIs can shift proximal mechanisms linked to adolescent mental health, including hopelessness and agency, and in some cases reduce depressive symptoms months later. For example, in a large trial of adolescents with elevated depressive symptoms, two online SSIs reduced depressive symptoms at 3 months relative to a supportive control, while also improving hopelessness and agency (Schleider et al., 2022). Similarly, a recent megastudy of 12 digital SSIs for depression found broad immediate mental health benefits across interventions, with a subset of SSIs yielding sustained depression symptom reductions; specifically, the two SSIs that retained specific design features common to effective SSIs improved symptoms across the 4‐week follow‐up (Kaveladze et al., 2026b). Together, these findings suggest that, if intentionally designed, immediate responses to high‐risk moments can have lasting mental health benefits. Generic referrals alone may be insufficient to motivate constructive action; instead, youth may feel motivated to engage when support is embedded where they already are—delivered in ways that are low‐barrier, private, and available without interpersonal disclosure.

If an adolescent expresses hopelessness, self‐criticism, panic, or isolation to a chatbot, that moment should trigger neither a refusal script nor a sustained, unpredictable, pseudo‐therapeutic conversation with an agent. It could instead trigger an offer of a brief, structured, evidence‐based intervention outside of the chatbot loop. That is, properly timed SSIs may help realize conversational AI's value as a bridge while reducing the risks associated with open‐ended, pseudo‐therapeutic chatbot interactions (e.g. chatbots attempting to simulate formal therapy). In this vein, conversational AI could be intentionally leveraged to identify moments of need and connect adolescents to supports whose content can be reliably predicted and tested. Evidence‐based digital single‐session interventions are especially well‐suited to this role because they are brief, bounded, and designed to target specific motivational and emotional processes (Dobias et al., 2022; Cohen et al., 2023). Other supports with these features may play similar roles in optimizing the safety and promise of genAI agents to promote youth mental health and help‐seeking.

Conversational AI is already part of adolescents' mental health support ecosystems. In its current forms, it can expose young people to real risks. It can also serve as a first point of contact when no other support is available. Companies and regulators have an ethical responsibility to design these systems for public benefit. For adolescents, that means designing conversational AI to become the safest, most constructive versions of the touchpoints they have already become, equipped to recognize and respond safely to distress and to connect youth to immediate, evidence‐based support and human care when needed.

## Conflict of interest statement

JLS serves on the Scientific Advisory Board for Walden Wise and the Clinical Advisory Board for Koko (unpaid) and receives book royalties from New Harbinger, Oxford University Press, and Little, Brown Book Group. JLS is co‐founder and chief scientific advisor for Navi. No Navi products were used or are referenced in the present manuscript. Koko (of which RM is CEO) has paid partnerships with OpenAI and Character.AI aimed at supporting users' mental health. Both authors (JLS and RM) collaborated on and were co‐authors on some of the research described and cited in this article.

## Ethics statement

No ethics approval was required for this debate article.

## Data Availability

Data sharing not applicable to this article as no datasets were generated or analysed during the current study.
